# Discovery and Characterization of an Aberrant Small Form of Glycoprotein I of Herpes Simplex Virus Type I in Cell Culture

**DOI:** 10.1128/spectrum.02659-21

**Published:** 2022-03-29

**Authors:** Xixi Gui, Wuchao Zhang, Peng Gao, Yongning Zhang, Lei Zhou, Xinna Ge, Xin Guo, John W. Wills, Jun Han, Hanchun Yang

**Affiliations:** a Key Laboratory of Animal Epidemiology of the Ministry of Agriculture and Rural Affairs, China Agricultural University College of Veterinary Medicine, Beijing, People’s Republic of China; b Department of Microbiology and Immunology, Pennsylvania State University College of Medicine, Hershey, Pennsylvania, USA; Oklahoma State University, College of Veterinary Medicine

**Keywords:** aberrant gel migration, cell-to-cell spread, cell-cell fusion, glycoprotein I, herpes simplex virus

## Abstract

The 380-to-393-amino-acid glycoprotein I (gI) encoded by herpes simplex virus 1 (HSV-1) is a critical mediator for viral cell-to-cell spread and syncytium formation. Here we report a previously unrecognized aberrant form of gI in HSV-1-infected cells. Production of this molecule is independent of cell type and viral strains. It had an unexpected gel migration size of approximately 23 kDa, was packaged into viral particles, and could be coimmunoprecipitated by antibodies to both N and C termini of gI. Deep sequencing failed to detect alternative RNA splicing, and the *in* *vitro* transcribed full-length mRNA gave rise to the 23 kDa protein in transfected cells. Combined mass spectrometry and antibody probing analyses detected peptide information across different regions of gI, suggesting the possibility of a full-length gI but with abnormal migration behavior. In line with this notion, the HA insertion mutagenesis revealed a stable fold in the gI extracellular region aa.38-196 resistant to denaturing conditions, whereas small deletions within this region failed the antibodies to detect the fast, but not the slow-moving species of gI. It is also intriguing that the structure could be perturbed to some extent by a gBsyn mutation, leading to exposure or shielding of the gI epitopes. Thus, the HSV-1 gI apparently adopts a very stable fold in its natural form, rendering it an unusual biophysical property. Our findings provide novel insight into the biological properties of HSV gI and have important implications in understanding the viral spread and pathogenesis.

**IMPORTANCE** The HSV-1 gI is required for viral cell-to-cell spread within the host, but its behavior during infection has remained poorly defined. Along with the classic 66 kDa product, here we report a previously unrecognized, approximately 23 kDa form of gI. Biochemical and genetics analyses revealed that this molecule represents the full-length form of gI but adopts a stable fold in its extracellular domain that is resistant to denatured conditions, thus contributing to the aberrant migration rate. Our results revealed a novel property of HSV-1 gI and have important implications in understanding viral pathogenesis.

## INTRODUCTION

Herpes simplex virus (HSV), an enveloped double-stranded DNA virus and the prototype of herpesviruses, has remained an important public health threat ever since its first isolation in the early 1900s but with no vaccines yet available (1, 2). This opportunistic agent is highly prevalent in human population and is often associated with various diseases, such as cold sore, keratitis, herpes encephalitis, neonatal herpes, and Alzheimer’s diseases, leading to a decreased life quality (3–7). HSV utilizes cell-to-cell spread (CCS) for transmission *in vivo*, a prominent feature that is highly relevant to viral pathogenesis (8). This transmission mode is essential for retrograde spread to establish latent infections in trigeminal ganglia and is also critical for anterior spread along axons to original infection sites during reactivation (9, 10).

The molecular machinery for HSV CCS is complex and requires the viral core fusion machinery (gB, gC, and gH/L) and a variety of other accessory factors (i.e., gE, gI, gK, etc.) that are not essential for the entry of extracellular virions (11–14). Among these proteins, the focus of this report is the envelope glycoprotein gI. This protein is well known to interact with gE to form the Fc receptor for immune evasion purposes (15–18). Although gI itself does not bind to IgG, its presence can dramatically enhance the affinity of gE with antibodies (19). This heterodimer also participates in HSV secondary development; simultaneous deletion of both can lead to severe budding defect (20). The role of gE/gI in HSV CCS is also well established (21, 22). It is currently clear that gE/gI is actively involved in sorting the nascent virions to the lateral cell junctions, but it remains elusive as to how the complex promotes nascent vision to come across the cell junction, striking a strong need to understand further their individual biological properties (23–25).

HSV-1 gI is a type I membrane protein that consists of a signal peptide (SP), an extracellular domain (ECD), a transmembrane domain (TM), and a C-terminal cytoplasmic tail (CT) (19, 21). It is unique in polymorphism among alpha-herpesviruses (10); it contains several tandem repeats (TR) in the sequence of the extracellular domain adjacent to the transmembrane region (26). This feature determines its size variations among different HSV strains (26). Most recently, we discovered a novel property of HSV-1 gI. That is, this protein possesses the ability to induce membrane curvature that gives rise to rod-shaped structures in both transiently expressed and HSV-infected cells (27). Further mutagenesis studies showed that this property of gI is critical for HSV syncytium formation in the UL24 syn background (27). In this report, we further explored the biological property of gI in HSV-1 infected cells. Unexpectedly, we discovered a previously unrecognized small form of gI that had an aberrant migration size of about 23 kDa, a molecular weight that is way much smaller than that by bioinformatics prediction. We subsequently investigated its origin and biological properties as described below.

## RESULTS

### Discovery of a 23 kDa species of HSV-1 gI in virus-infected cells.

**FIG 1:**
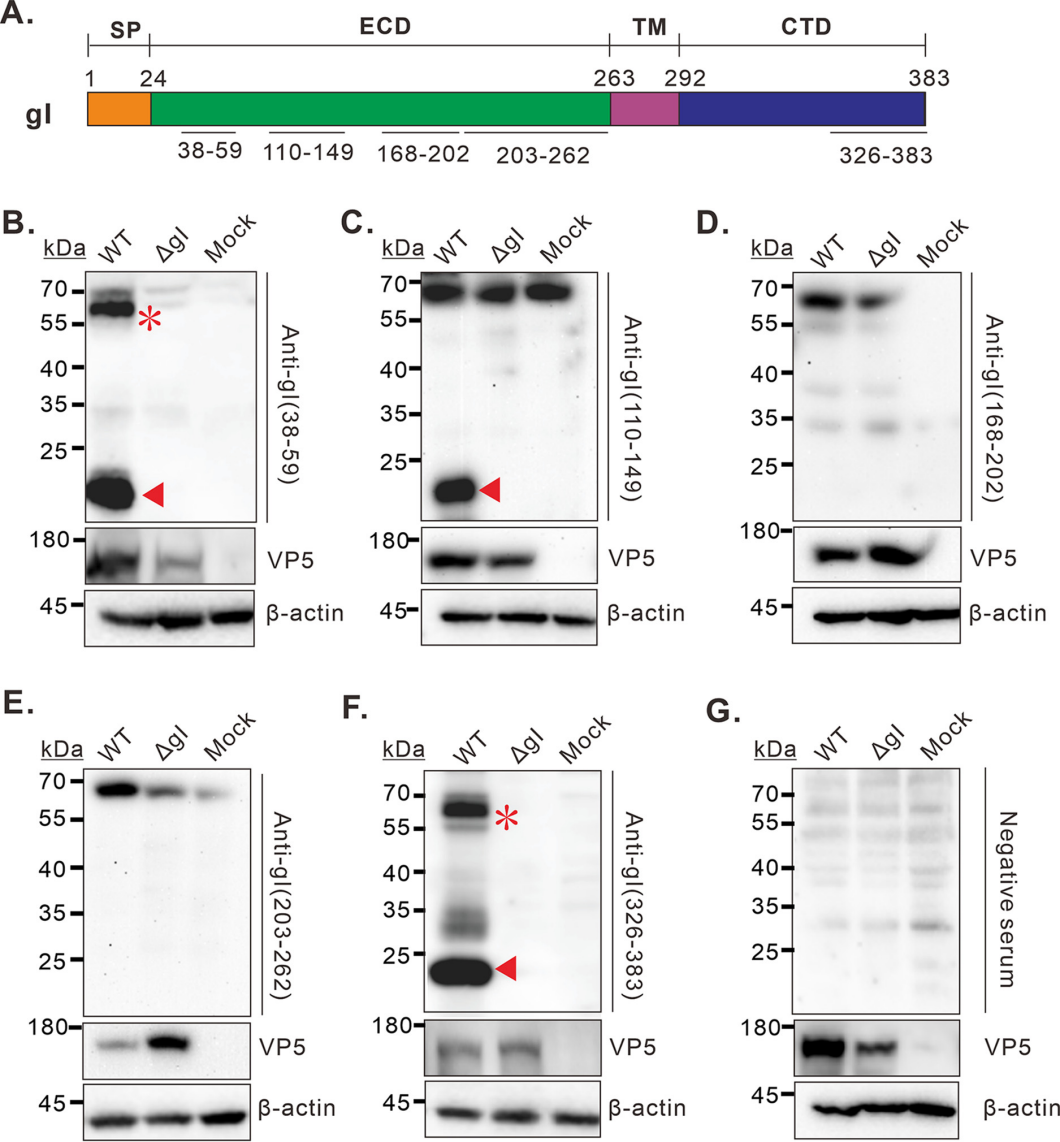
Discovery of a 23 kDa species of gI in HSV-1 infected cells. (A) Diagram of the domain organization of HSV-1 KOS strain gI and the recognition sites of gI polyclonal antibodies. (B–G) Vero cells were mock infected and infected with HSV-1 strain KOS (WT and ΔgI) at an MOI of 5. At 24 hpi, the cells were harvested and lysed for immunoblotting analysis with indicated antibodies. The major migration forms of gI were marked by asterisk and triangle at corresponding sites.

We have recently discovered an unusual property of HSV-1 gI, namely, that it can induce rod-shaped structures in both transfected and infected cells as detected by immunofluorescence assay (IFA) (27). However, how HSV-1 gI behaves under reducing conditions has remained not fully explored. For this purpose, we have made five rabbit peptide antibodies against different regions of gI as indicated in Fig. 1A. These antibodies were named as anti-gI(38-59), anti-gI(110-149), anti-gI (168-202), anti-gI(203-263), and anti-gI(326-383), respectively. They all recognized well gI-GFP in transfected BHK-21 cells and the native gI in HSV-1 infected cells by IFA (supplemental data Fig. S1 in the supplemental material).

To further investigate the biological property of HSV-1 gI, Vero cells were either mock-infected with DMEM or infected with WT HSV-1 KOS strain or its derivative, namely, the gI-deletion mutant (HSVΔgI) at an MOI of 5. At 24 hpi, Vero cells were harvested, and the cellular proteins were separated by SDS-PAGE, transferred to a nitrocellulose membrane, and probed with the antibodies as indicated. Two antibodies anti-gI(38-59) and anti-gI(326-383) worked well (Fig. 1B and F). They could recognize the classic 66 kDa product that reportedly represents the full-length mature gI (28). Unexpectedly, they also reacted with a fast-moving species with a molecular weight of approximately 23 kDa (Fig. 1B and F). This small product has never been reported before, and the migration rate was much lower than the predicted full-length gI by bioinformatics (about 40 kDa). Moreover, this species was specific to gI, as the corresponding band was missing in either mock-infected (Fig. 1B and F, lane 3) or HSVΔgI-infected Vero cells (Fig. 1B and F, lane 2).

The other three peptide antibodies [anti-gI(110-149), anti-gI(168-202), and anti-gI(203-263)] somehow reacted with a band with a size similar to the 66 kDa product in the control groups for unknown reasons (Fig. 1C to E), raising the doubt of the specificity. However, it should be mentioned that the antibody anti-gI(110-149) could specifically recognize the 23 kDa species (Fig. 1C). In any case, it is clear that HSV-1 expresses a 23 kDa form of gI with unknown origin during infection, and this product contains at least the N and C terminal portions of gI.

### Cell-type and viral-strain independent production of the 23 kDa gI molecule.

**FIG 2:**
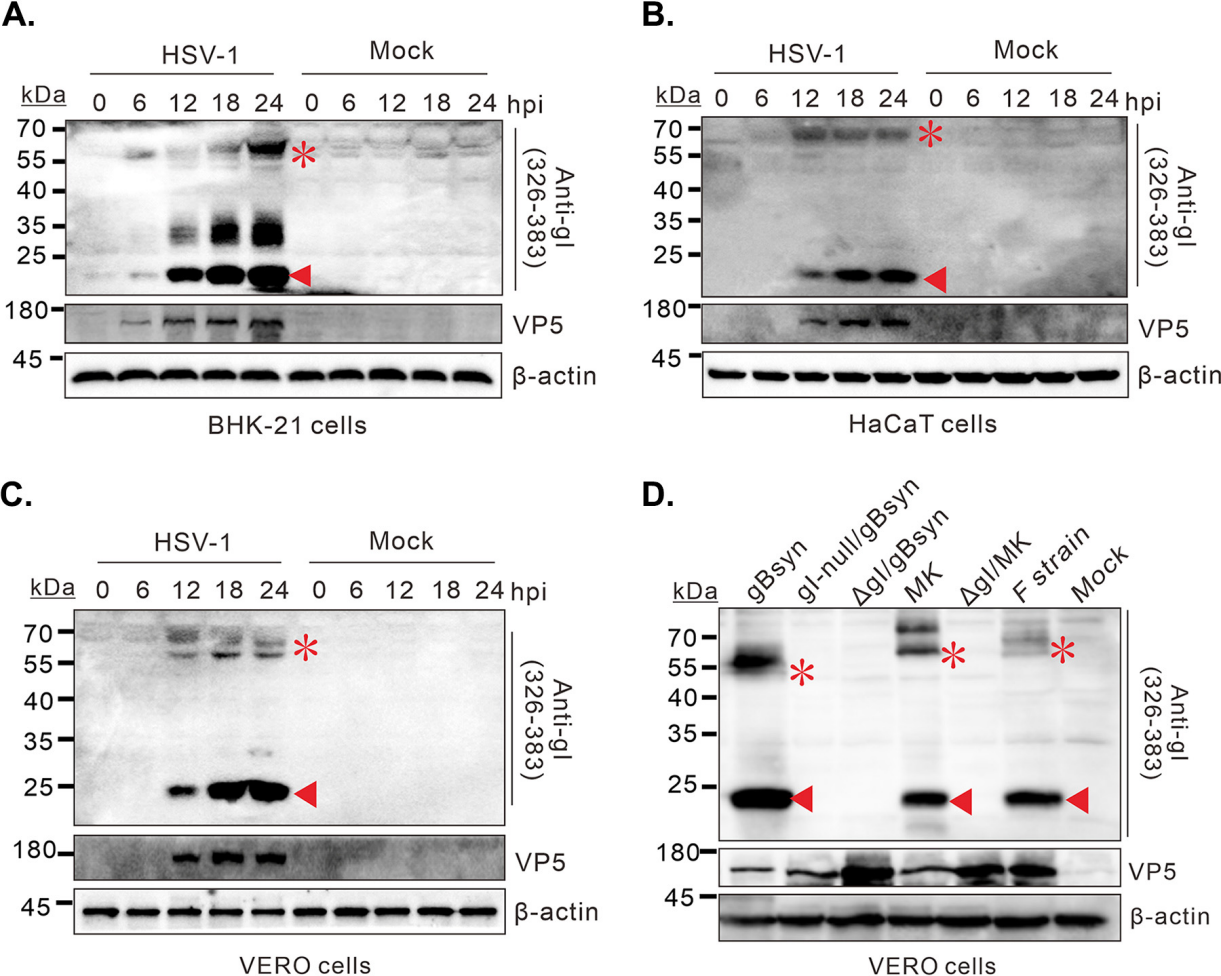
Production of the 23 kDa gI species is independent of cell type and viral strains. (A) BHK-21, (B) HaCaT, and (C) Vero cells were either mock-infected or infected with HSV-1 strain KOS at an MOI of 5. The cells were harvested at the indicated times and viral or cellular proteins were analyzed by Western blotting with indicated antibodies. (D) The same as above, except that different viruses were used.

To test the cell-type dependence, BHK-21 (Fig. 2A), HaCaT (Fig. 2B), and Vero cells (Fig. 2C) were infected with HSV-1 KOS strain at an MOI of 5 and harvested at indicated time points for Western blot analysis. The 23 kDa gI was expressed in all tested cell types and arose about the same time as the 66 kDa band at the late stage of HSV-1 life cycle. Moreover, the 23 kDa band had a much higher abundance than the 66 kDa form. Occasionally, we could observe a species with a 25-to-35-kDa molecular weight. We also investigated the gI expression in the cells infected with different strains (Fig. 2D), including HSV-1 KOS strain, gBsyn (KOS background), F strain, and McKrae strain. We engineered the corresponding mutants lacking the gI-coding region as the negative control. In all cases, the 23 kDa species was produced, but not in the cells infected with the gI-deletion mutants (Fig. 2D). Thus, the expression of the fast-moving gI species is independent of cell types or viral strains.

### HSV-1 gI mutant defective of induction of rod-shaped structures retains the ability to make the 23 kDa species.

**FIG 3:**
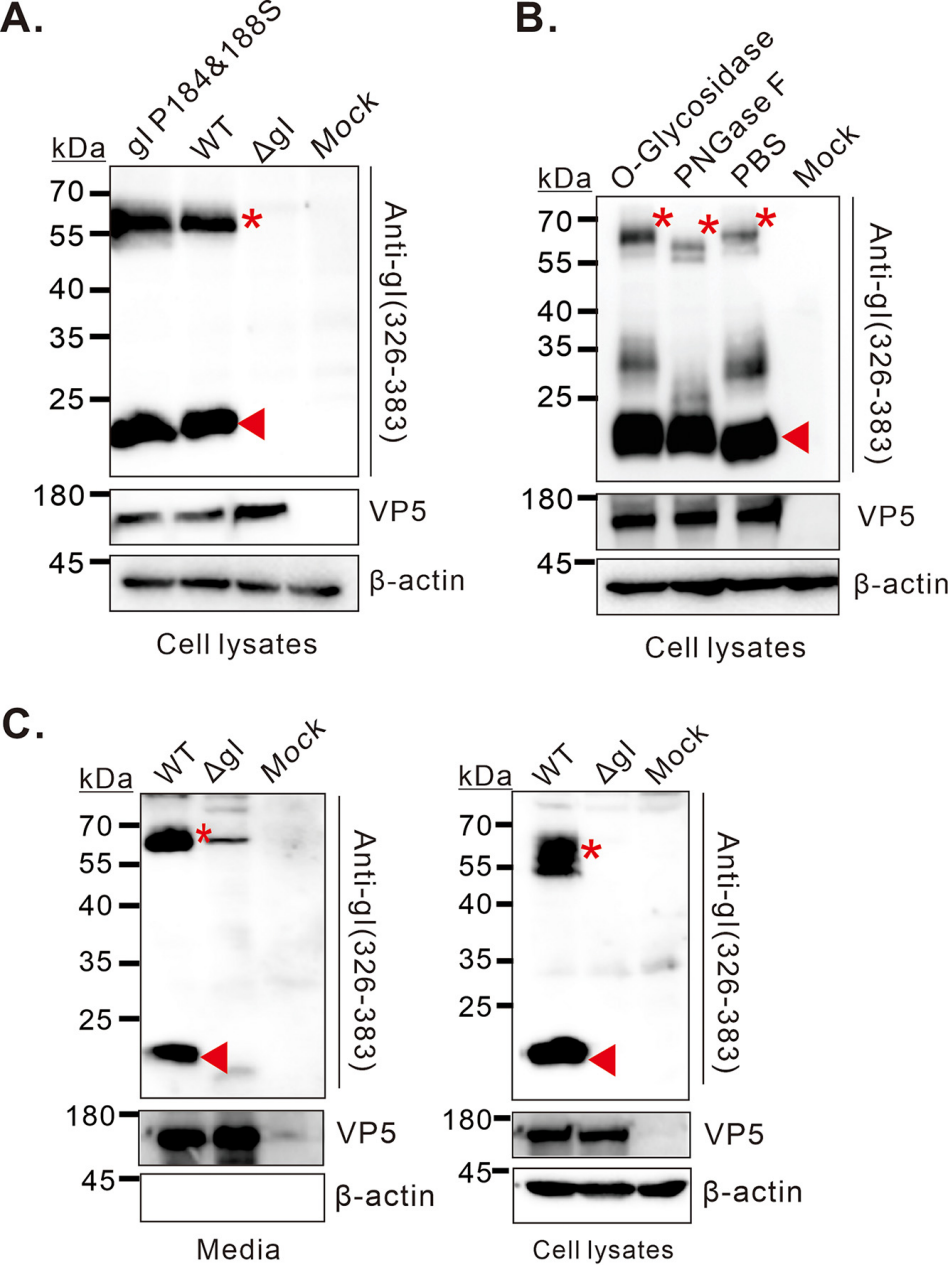
The 23 kDa gI likely represents the immature form and is packaged into virion particles. Vero cells were infected with indicated viruses at an MOI of 5. At 18 hpi, the cells or supernatant were harvested for different assays. (A) Western blot analysis of the mutational effect of gI P184&188S on production of the fast-moving species. (B) Effect of the glycosidase treatment on the gI gel migration. (C) Virion packaging analysis of the 23 kDa species. Virions in the supernatants were purified and analyzed by Western blotting with indicated antibodies (left panel). The right panel shows the intracellular expression of gI. Capsid protein VP5 and β-actin were used as the control.

We have recently shown that the HSV gI induces in mammalian cells rod-shaped structures, a higher-order form of gI (27). We also mapped the critical residues to the P_184_XXXP_188_ motif, and the mutant gI P184&188S fails to induce rod-shaped structures in either transfected or infected cells (27). We hypothesized that this property might affect the gI migration rate in SDS-PAGE. Unfortunately, this was not the case. The 23 kDa product could still be detected in the cells infected with the mutant P184&188S (Fig. 3A). Thus, the aberrant gel migration behavior of HSV-1 gI is not relevant to its ability to induce membrane curvatures.

### The 23 kDa gI likely represents the immature form and is packaged into virion particles.

Previous studies have shown that the HSV gI contains O or N-linked glycosylation sites (29, 30). To investigate whether the gI small molecule is modified by glycosylation, the HSV-infected cell lysates were treated with two different endoglycosidases (PNGase F and O-Glycosidase) to remove the respective N and O-linked glycans, and then analyzed by Western blotting. The results showed that treatment with PNGase F led to a shift of the 66 kDa band (Fig. 3B, lane 2), but not by the treatment with O-Glycosidase (Fig. 3B, lane 1), suggesting that the gI undergoes N-linked glycosylation in virus-infected cells. However, in both cases, the treatment did not have affect the mobility of the 23 kDa product, suggesting that the small molecule likely represents the immature form of gI.

Vero cells were infected with the WT or ΔgI at an MOI of 5 to determine whether the small form of gI can be packaged into HSV virions. At 18-h postinfection, the supernatants of infected cells were harvested, pelleted through a 30% sucrose cushion, and analyzed by SDS-PAGE and Western blot with antibodies against gI. The antibodies to VP5 served as a loading control. Interestingly, both forms were detected in virion particles (Fig. 3C, left panel). As a negative control, β-actin was not detectable in the purified virions (Fig. 3C, left panel), but could be detected in cell lysates (Fig. 3C, right panel).

### The 23 kDa gI is not a degraded product.

**FIG 4:**
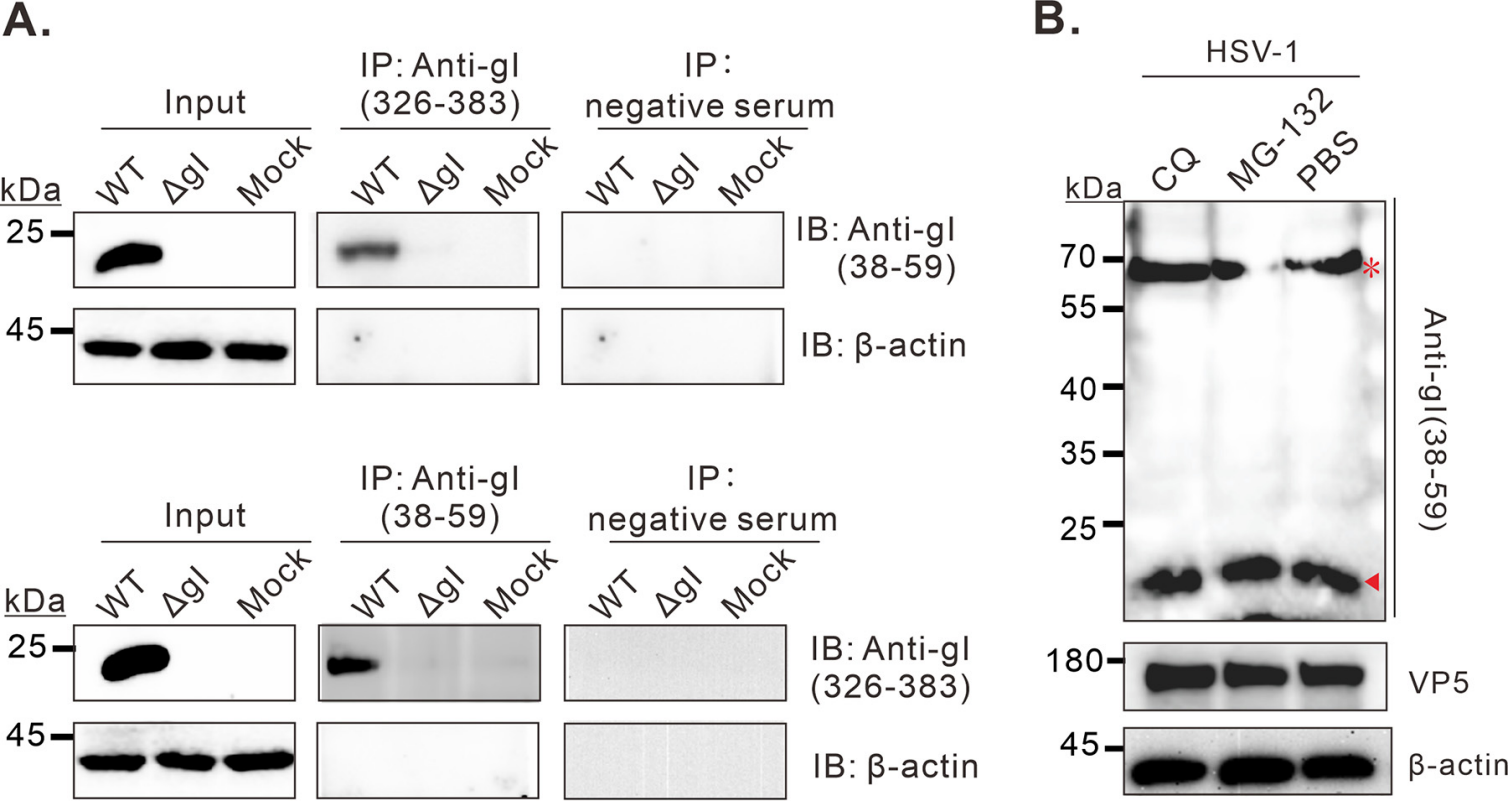
The 23 kDa gI is not a degraded product. Vero cells were mock infected with DMEM or infected or with the indicated viruses at an MOI of 5. (A) At 24 hpi, the cells were lysed by NP40 buffer, and then the supernatants were harvested for co-immunoprecipitation assay with the indicated antibodies. (B) Otherwise, at 12 hpi, the cells were incubated with CQ (20 μM/mL) or MG132 (20 μM/mL) for 12 h and then analyzed by Western blotting with anti-gI(38–59).

To rule out the possibility of protein degradation, we first performed a co-immunoprecipitation (IP) assay with the antibodies against the N- or C termini of gI. If the 23 kDa product happens to be a mixture of two different molecules with identical molecular weight, the Co-IP assay coupled with Western blot analysis will be able to tell the difference. Discouragingly, the 23 kDa product could be pulled down with either antibody [anti-gI(326-389) and anti-gI (38-59)] from HSV-1 infected cells (Fig. 4A). In a second assay, the infected or mock-infected cells were incubated with MG-132 (a proteasome inhibitor) or chloroquine (CQ, a lysosomal inhibitor) for 12 h to inhibit protein degradation via proteasome and lysosome pathways (Fig. 4B), the most common pathways for protein degradation. Further Western blot analysis with anti-gI (38-59) was used to the production of the small molecule. Again, treatment with both drugs did not appreciably affect the accumulation of the small form of gI (Fig. 4B). Thus, these results suggest that the small form is less likely a degraded product.

### The 23 kDa product is not a result of alternative splicing at the pre-mRNA level.

**FIG 5:**
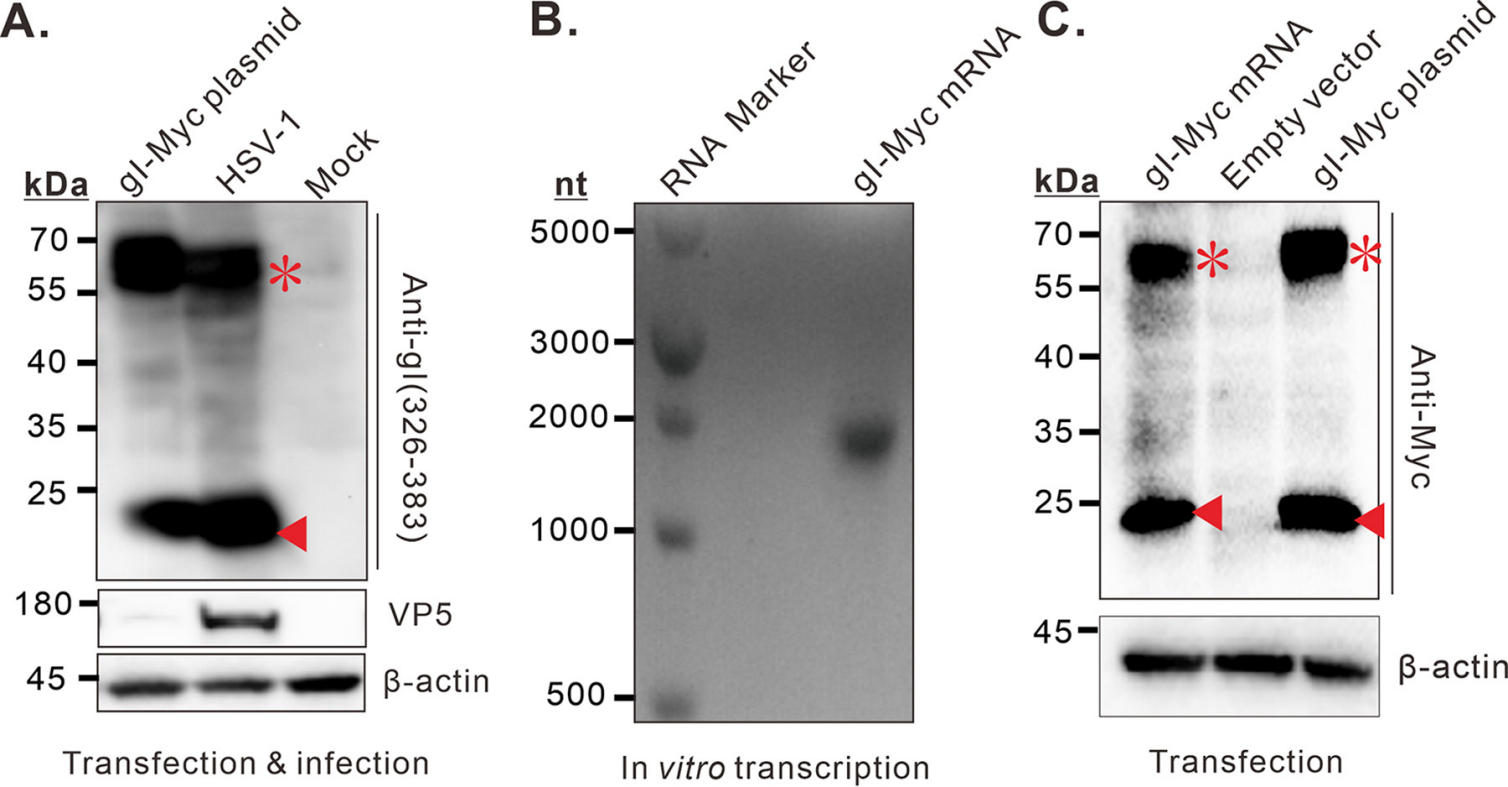
The 23 kDa product is not a result of alternative splicing at RNA level. (A) BHK-21 cells were transfected with gI plasmid or infected with HSV-1 KOS strain. At 24 hpi or transfection, the expression of gI was analyzed by immunoblotting with anti-gI (326-383). (B) The *in vitro* transicribed, purified gI mRNA was analyzed by 1% agarose gel electrophoresis. (C) Transient expression of *in vitro* transcribed gI-Myc mRNA in BHK-21 cells.

It is also possible that gI is a spliced product at RNA level. To test this hypothesis, we carried out two different experiments. In the first assay, we performed a transcriptomic analysis of HSV-1 infected cells. Deep sequencing was used to detect possible alternative mRNA splicing. The result showed that there were a lot of alternative mRNAs splicing events in HSV-1 infected cells, but no alternative splicing of viral gI mRNA was found (data not shown), which was also confirmed by real-time PCR (data not shown). In a second assay, we performed *in vitro* transfection assay by transfecting a eukaryotic plasmid encoding gI-myc. We found that the transfected cells could express both the 23 kDa and 66 kDa products (Fig. 5A), suggesting that production of the 23 kDa form does not require any other viral factors. To further rule out the possibility of alternative splicing, we *in vitro* transcribed gI mRNA using a T7 RNA polymerase transcription system. The capped gI-Myc mRNA was examined in agarose gel electrophoresis (Fig. 5B) and then transfected into BHK-21 cells. Again, we were able to detect the 23 kDa product (Fig. 5C), ruling out the possibility of alternative RNA splicing.

### The HSV-1 gI adopts a stable fold that can be perturbed by gBsyn mutation.

We performed two experiments to rule out the possibility that the 23 kDa form is a spliced product at protein level. In the first assay, we performed mass spectrometry analysis. The 23 kDa product was Co-IP by anti-gI(326-383), separated by SDS-PAGE and silver-stained. The gel band corresponding to the small gI was cut and sent out for mass spectrometry analysis. The MS analysis identified the peptides aa.88-133, 140-148, 186-193, and 331–344 (Fig. S2 in the supplemental material). This result is consistent with the antibody probing analysis as described above (Fig. 1).

**FIG 6:**
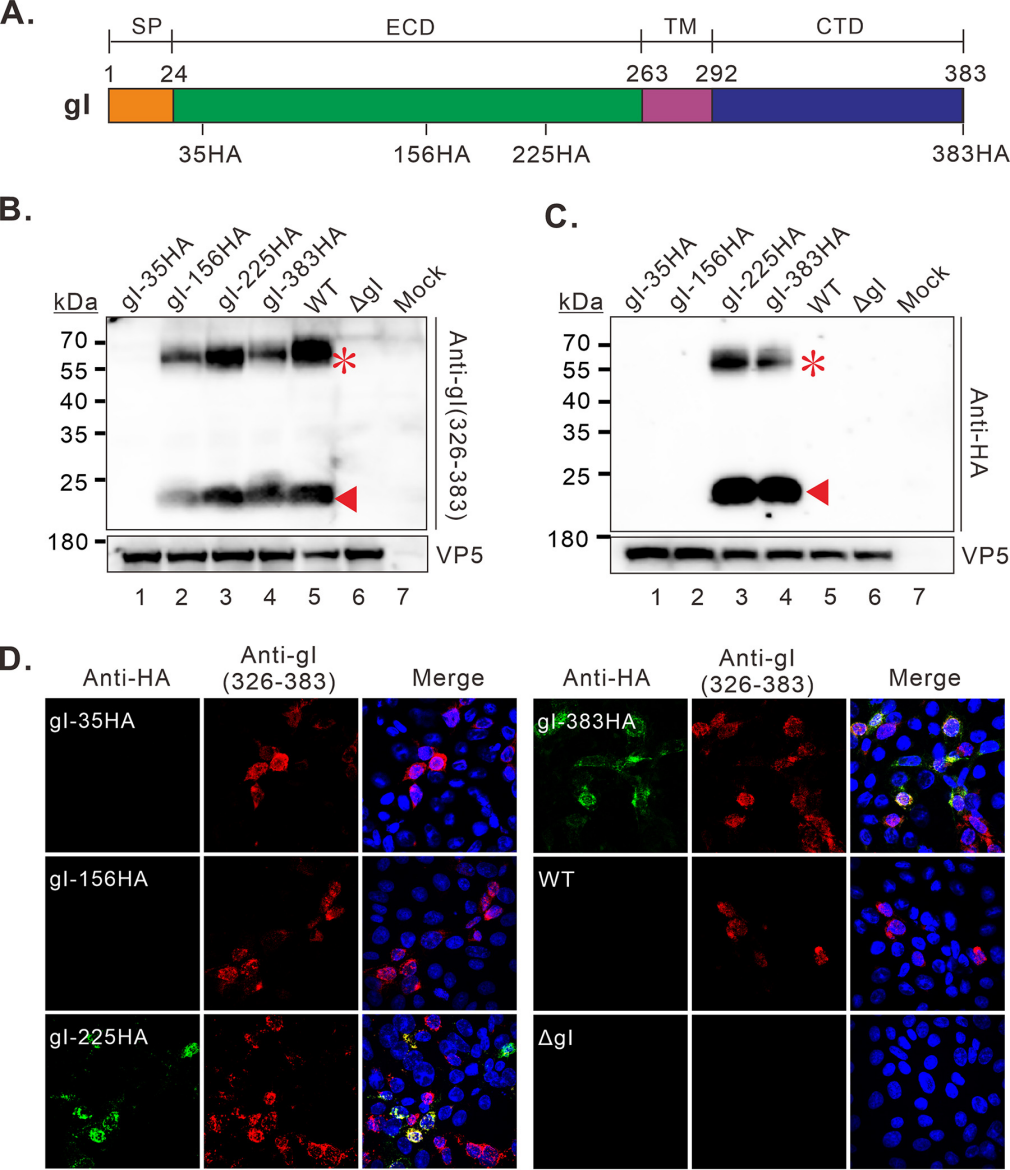
The HSV-1 gI adopts a stable fold. (A) Indication of the positions of HA-tag insertion within HSV-1 gI. (B–C) Western blot analysis of gI production in Vero cells infected with indicated viruses in the background of WT KOS at 24 hpi. (D) Immunofluorescence analysis of gI expression in Vero cells at 18 hpi with indicated antibodies. The representative images were captured with a Nikon A1 confocal microscope (100X objective) and further processed using Image J.

As the peptide antibodies to the gI middle region did not work well for Western blot (Fig. 1), we performed insertion mutagenesis by inserting the HA epitope into the different locations of gI (Fig. 6A). Consequently, a series of recombinant viruses were engineered in the background of both WT KOS and gBsyn. These mutants were confirmed by DNA-sequencing of the gI-coding region. The phenotype of the recombinant viruses was analyzed by immunoblot and IFA. We found that the antibody anti-gI (326-383) could detect both 66 kDa and 23 kDa products in Vero cells infected with all the recombinant viruses excect the mutant gI-35HA (Fig. 6B). In contrast, the antibodies to HA could only recognize the gI products in cells infected with the mutants gI-225HA and gI-383HA (Fig. 6C). The same was true for the IFA assay (Fig. 6D). Thus, these results suggest that the amino acids near position 225 are part of the gI small molecule, and that the HSV-1 gI region aa38-200 adopts a usual stable fold that is resistant to denatured conditions, leading to shielding of the epitopes near position 38 and 156 inside the structure.

**FIG 7:**
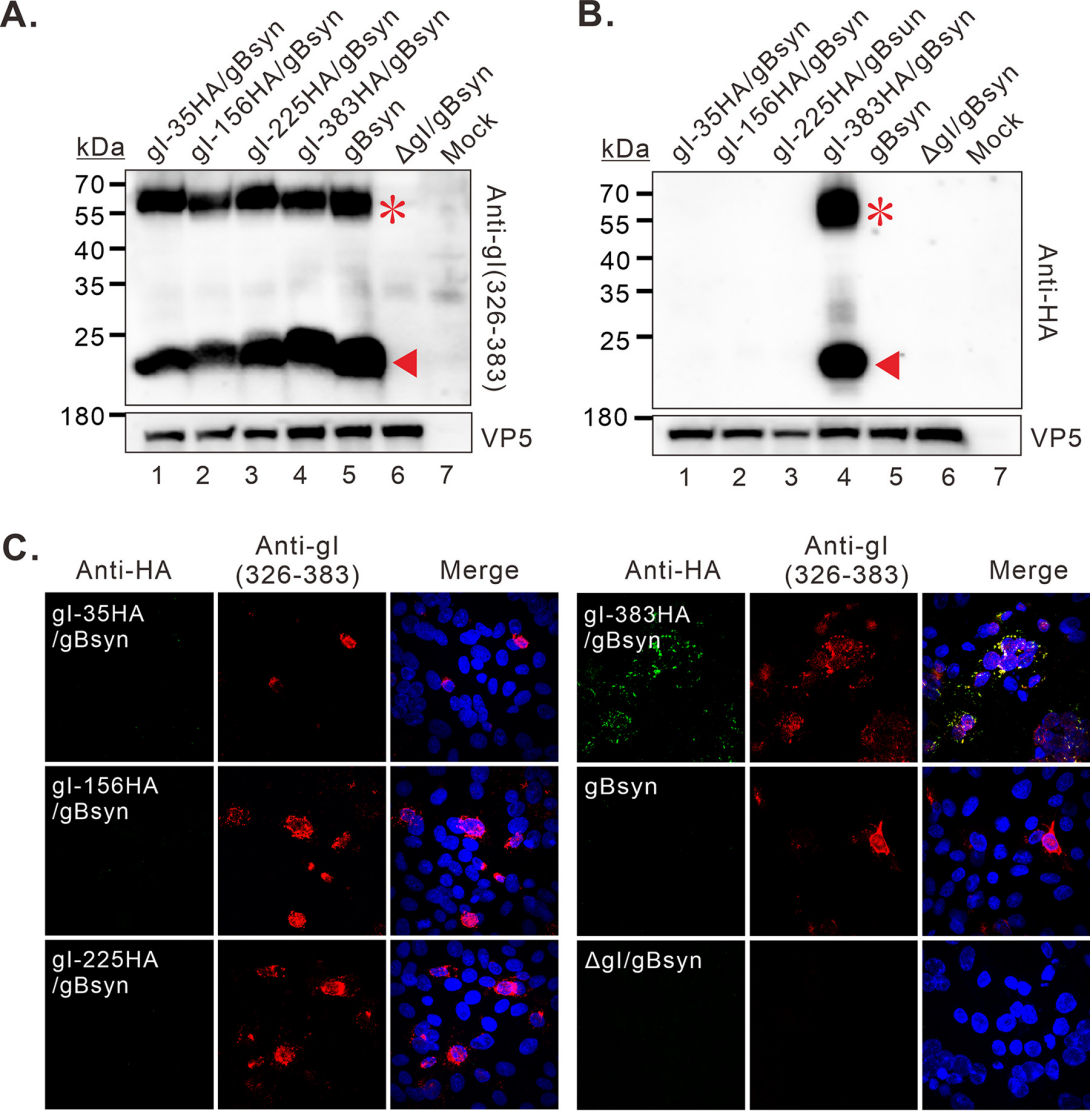
The effect of gBsyn mutation on the gI recognition by different antibodies. (A, B) Western blot analysis of gI production in Vero cells infected with indicated viruses in the gBsyn background at 24 hpi. (C) Immunofluorescence analysis of gI expression in Vero cells at 18 hpi with indicated antibodies. The representative images were captured with a Nikon A1 confocal microscope (100X objective) and further processed using Image J.

Interestingly, under the background of gBsyn, the antibody anti-gI (326-383) could detect both 66 kDa and 23 kDa products in Vero cells infected with all the recombinant viruses (Fig. 7A). However, this time, the anti-HA antibodies failed to detect the gI products in gI-HA225/gBsyn infected cells (Fig. 7B, lane 3), compared to the WT background (gI-HA225) (Fig. 6C, lane 3). The IFA analysis showed the same results (Fig. 7C). Thus, these results suggest that the gBsyn mutation can affect gI structure via a yet understood mechanism and results in perturbation of gI local structure in both the extracellular domain and the cytoplasmic tail, leading to the epitopes either buried (position 225) or exposed (position 35). Thus, gI exhibits different structures in gBsyn and wild-type background. Collectively, the above results indicate that the 23 kDa product most likely represents the full-length gI protein but adopts a stable structure resistant to reducing conditions.

### Mapping the critical regions for the aberrant migration behavior.

**FIG 8:**
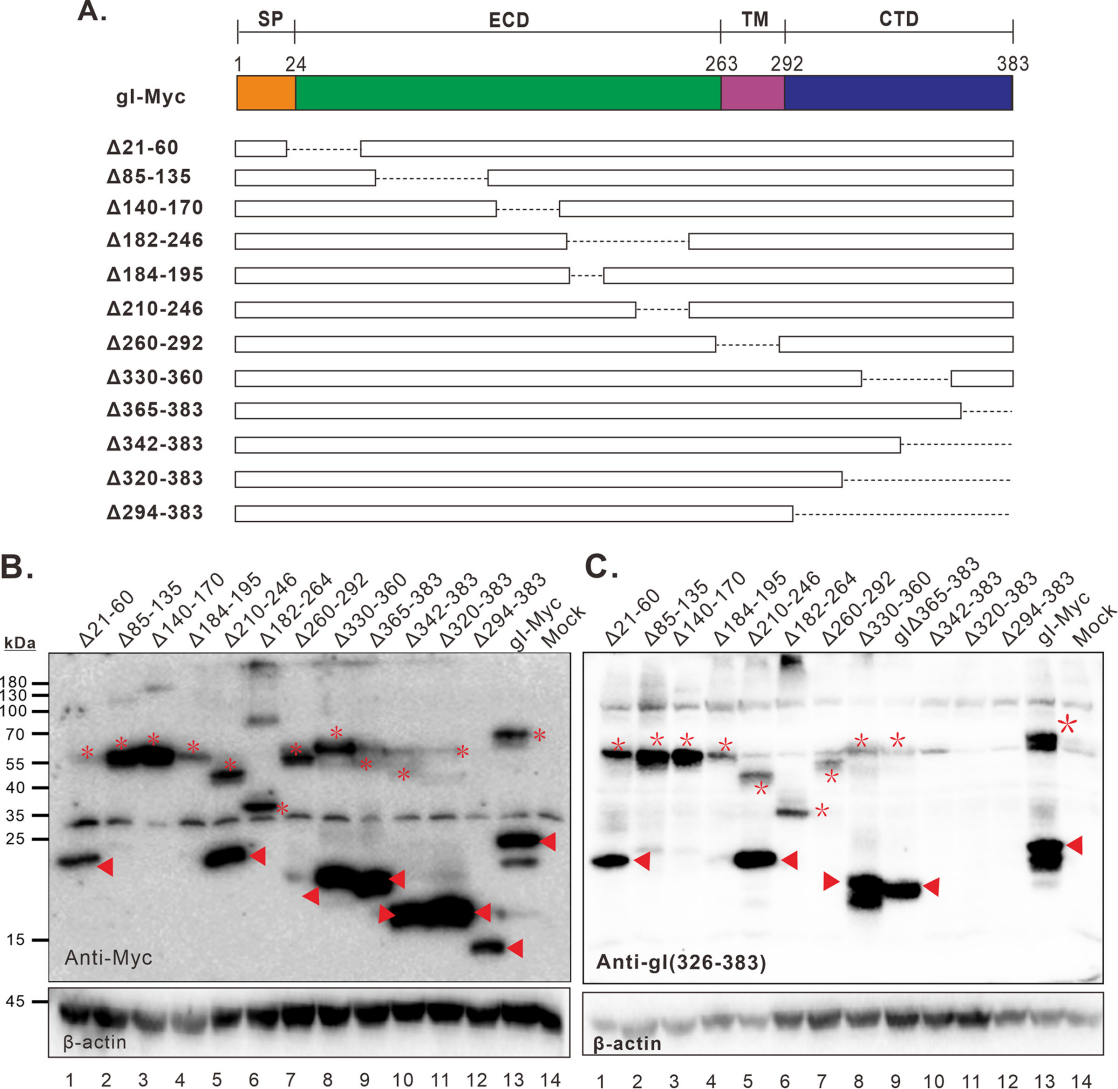
Mapping the critical regions for the aberrant migration behavior of HSV-1 gI. (A) Construction of gI deletion mutants based on the plasmid coding for gI-Myc. (B and C) BHK-21 cells were transfected to express either WT gI or the mutants. At 24 h posttransfection, the cells were harvested and lysed for immunoblotting with indicated antibodies anti-Myc antibody (B) or anti-gI(326-383) (C). Different migration forms of gI were marked by asterisk or triangle at corresponding sites.

If the fast-moving property of gI is attributed to the special structure, then small deletions within this region should perturb the mobility of gI in the denaturing SDS-PAGE gel. To test this hypothesis, we created a series of deletion mutants across the whole gI-coding region based on the plasmid coding for gI-Myc as indicated in Fig. 8A, in which the c-Myc epitope is placed at the C-terminus. The transient expression assay showed that deletion of the cytoplasmic tail (gIΔ330-360, gIΔ365-383, gIΔ342-383, gIΔ320-383, and gIΔ294-383) led to expression of smaller products moving faster than the 23 kDa species (Fig. 8B and C, lanes 8–12, respectively). As expected, the antibody anti-gI (326-383) failed to detect gI in the cells expressing gIΔ342-383, gIΔ320-383, and gIΔ294-383 due to deletion of the relevant epitopes (Fig. 8C, lanes 10–12). Meanwhile, a migration shift was also observed for slower-moving species compared to the 66 kDa band. Thus, these results suggest that the cytoplasmic tail is not the determinant for the aberrant migration rate of gI. In contrast, deletions within the gI region aa.60-200 (gIΔ85-135, gIΔ140-170, and gIΔ184-195) led to undetectable level of the fast moving species even in the presence of MG132 (Fig. 8B and C, lanes 2–4, respectively). However, the deletions did not affect the detection of the slower-moving species, suggesting that the region aa.60-200 is critical for the abberent behavior. The region near TM was not essential, as deletion of the gI aa.210-246 did not cause much shift of the 23 kDa species, but rather resulted in a significant shift of the 66 kDa species (Fig. 8B and C, lane 5). In line with these, a further deletion into the region aa.60-200 (gIΔ182-264) led to the disappearance of the 23 kDa species but allowed the detection of a 35 kDa-or-so species and a product over 70 kDa (probably the dimer) (Fig. 8B and C, lane 6). Finally, deletion of the transmembrane region (TM, 260-292) also led to disappearance of the fast-moving species (Fig. 8B and C, lane 7). Overall, the above results suggest that the gI region aa.60-200 and TM contribute to the aberrant migration behavior of HSV-1 gI in the gel.

## DISCUSSION

Despite the well-established roles of HSV-1 gI in viral pathogenesis, the biological property and how it executes its function have remained poorly understood (16, 27, 31). This study discovered a previously unrecognized 23 kDa species of HSV-1 gI in virus-infected cells. We provided evidence to show that this small molecule is not a degraded or spliced product, but rather represents the full-length gI. We also showed that the HSV-1 gI apparently adopts a rather stable fold resistant to heat (boiling) and denaturing conditions (SDS). Intriguingly, the gBsyn mutation can somehow exert an effect on gI, leading to perturbation of its structure. The relevant significance and insights are discussed as below.

In retrospect, the mature form of HSV-1 gI was reported to be a product of 66–68 kDa in size that can be recognized by monoclonal antibody Fd69 and polyclonal antibodies (15, 32–37). In the absence of N-linked carbohydrates, the product has a molecular weight of about 58 kDa (33). This is considerably larger than that from the *in vitro* translated gI mRNA (52-55 kDa). This report unexpectedly discovered a 23 kDa species of HSV-1 gI (Fig. 1), along with the 66 kDa band. This size is much smaller than the predicted immature form (40.7 kDa). The idea that the 23 kDa product is derived from alternative RNA splicing was quickly rejected, as both deep sequencing and RT-PCR failed to detect a spliced gI mRNA. More importantly, the *in vitro* transcribed gI mRNA directly led to the expression of the 23 kDa product once transfected into mammalian cells (Fig. 6). Since mRNAs generally do not travel back to the nucleus, where RNA splicing machinery resides, it is unlikely that the transfected gI mRNA would undergo further splicing in the cytoplasm (38–40). Meanwhile, the result suggests that the production of this small form is a property of gI itself and does not need other viral factors (Fig. 6).

The possibility of protein degradation was also ruled out. We showed that the 23 kDa product could be specifically recognized and co-immunoprecipitated by the rabbit antibodies to the N- or C termini of gI (Fig. 1 and 4). It is less likely that the 23 kDa product contains two fragments that have identical molecular weight but interact with each other (Fig. 4). If that is the case, the glycosidase treatment or deletion mutagenesis of gI will reveal two fragments with different sizes. Unfortunately, this was not the case (Fig. 3B and Fig. 8B). In addition, the drug inhibitors targeting the common protein degradation pathways (proteasome and lysosome) did not affect the accumulation of the small form of gI (Fig. 4B).

Protein splicing might serve as an attractive alternative explanation (41). It is a post-translational processing event that involves the precise removal of an internal polypeptide segment from a precursor protein with the concomitant ligation of the flanking polypeptide sequences (42–44). However, multiple pieces of evidence are against this idea. The combined mass spectrometry analysis and antibody probing (Fig. 1) of the 23 kDa gI detected peptides spreading across different positions of gI (aa.38-59, 88-133, 110-149, 186-193, and 326–383). Although the information regarding the region aa.203-263 is missing, further analysis by HA insertion mutagenesis confirmed the inclusion of this region (Fig. 6). On the other hand, we favor the idea that the HSV-1 gI adopts a stable fold. The evidence came from the HA insertion mutagenesis, in which anti-HA antibodies could recognize the HA epitope only at certain locations (gI-225HA and gI-383HA) but not others (gI-35HA and gI-156HA), whereas anti-gI (326-383) could detect the 23 kDa product in all cases except the mutant gI-35HA (Fig. 6). These results suggest that a stable local structure prevents recognition of the HA epitope at position 156 under denaturing condition. It is interesting to note that the HA insertion at position 35 affected the recognition of the small molecule by all the available antibodies [anti-gI(38-59), gI (323-383) and anti-HA antibody)], suggesting that the insertion has a profound effect on the overall structure of gI in a yet-unknown manner. In any case, it is clear that the HSV-1 gI adopts some kind of stable fold. It is conceivable that this stable structure may render gI refractory to various denaturing environments and likely provides a resilient platform for protein-protein interactions during virus spread across cell junctions. Future studies may be directed to investigate the role of this stable fold in HSV-1 pathogenesis by deletion or site-directed mutagenesis of gI extracellular domain.

Tertiary structure of membrane proteins has been reported to contribute to both detergent-loading levels and polypeptide-SDS-PAGE migration rates and have been linked to the anomalously fast migration under reducing conditions (45–48). Consistent with this notion, we showed that deletion of the stable fold-containing region failed the antibodies to detect the fast-moving species (Fig. 8). Thus, an altered detergent binding might explain the anomalous SDS-PAGE behavior of gI. Certainly, it is also possible that gI might have gone through some sort of modification such as lipidation, which may render a faster migration rate (49, 50).

Interestingly, the gBsyn mutation also exerted an unexpected effect on the gI structure (Fig. 7). Under the gBsyn background, the anti-HA antibody failed to detect the 23 kDa product in the cells infected with gI-HA225/gBsyn, that would otherwise be detected in the WT background (gI-HA225) (Fig. 6). Although the molecular details are not clear, it is conceivable that the gBsyn mutation might have altered the protein interactions among a complex network, including gI and gB, that eventually lead to a conformational change of gI. Future studies might be directed to unveil the underlying mechanisms, which might help understanding how gI works in the cell-cell spread.

## MATERIALS AND METHODS

### Cells and viruses.

Vero, BHK-21, and HaCaT cells were all cultured in Dulbecco’s modified Eagle’s medium (DMEM) (Gibco, catalog no. 12491015) with 10% fetal bovine serum (FBS) (Gibco, catalog no. 16140071) and penicillin (50 U/mL) & streptomycin (50 μg/mL) at 37°C in a humidified atmosphere of 5% CO_2_. The HSV-1 KOS strain, the genome of which has been cloned into a bacterial artificial chromosome, was provided by David Leib (Dartmouth College of Medicine, Hanover, NH) and has been described before (51). The HSV-1 F strain was provided by Professor Chunfu Zheng (Fujian Medical University, China), and the McKrae strain was a gift from Jumin Zhou (Kunming Institute of Zoology, Chinese Academy of Sciences, China). For infection assays, cells were grown in DMEM supplemented with 2% FBS, penicillin (50 U/mL), and streptomycin (50 μg/mL).

### Antibodies.

The rabbit peptide antibodies (anti-gI(38-59), anti-gI(110-149), anti-gI (168-202), anti-gI(203-263), and anti-gI(326-383) serum) have been described previously (27). Mouse anti-HA monoclonal antibody (MAb) (catalog no. 2367), mouse anti-Myc MAb (catalog no. 2276) and mouse anti-actin Mab (catalog no. 3700) were bought from Cell Signaling Technology (MA, USA). Rabbit anti-VP5 antibody was provided by Richard Courtney (Pennsylvania State University College of Medicine, Hershey, PA). Alexa Fluor 488-conjugated goat anti-mouse F(ab')_2_ fragment (catalog no. 11017), Alexa Fluor 488-conjugated goat anti-rabbit F(ab')_2_ fragment (catalog no. 11070), and Alexa Fluor 568-conjugate goat anti-rabbit F(ab')_2_ fragment (catalog no. 11011) were obtained from Thermo Fisher (CA, USA). Protease inhibitors cocktail (catalog no. P8340) and MG-132 (catalog no. S2619) were obtained from Sigma–Aldrich (MO, USA).

### Construction of gI mutants.

The mammalian plasmids coding for gI-myc or gI-HA was created by cloning HSV-1 KOS strain gI fused with a c-Myc or HA epitope tag at the C-terminus into the XhoI and BamHI sites in the vector pEGFP-N2. The molecular cloning was facilitated through homologous recombination by using the ClonExpress II one-step cloning kit (Vazyme, Nanjing, China). The corresponding deletion or site-directed mutants originated from gI-Myc were made by standard molecular biological techniques. All created plasmids were under the control of the cytomegalovirus promoter. A Kozak core sequence was also added to allow optimal expression and confirmed by DNA sequencing. For transient expression, BHK-21 cells seeded in six-well plates with coverslips were transfected when 60% to 70% confluent with 2.0 μg plasmid DNA per well using Lipofectamine 2000 by following the manufacturer’s instructions (Invitrogen, CA, USA).

### *In vitro* transcription.

The full-length DNA sequence of gI-Myc was amplified from the plasmid coding for gI-myc by DNA polymerase with specific primers, with a T7 promoter sequence (TAATACGACTCACTATAGG) at the 5’end, and a ploy(A) tail at the 3’end. Then, the gI mRNA was transcribed by using T7 RNA polymerase according to the protocol of the RiboMAX Large Scale RNA Production Systems—SP6 and T7 (Promega, catalog no. P1300). The *in vitro* transcribed gI mRNA was purified by Monarch RNA Cleanup Kit (New England Biolabs, catalog no. T2040L), verified in 1% agarose, and then used for subsequent transfection experiment.

### Construction of HSV-1 gI mutant viruses.

The HSV-1 gI P184&188S, gI-null, and ΔgI mutants, as well as HSV-1 KOS gBsyn have been described previously (27). The BAC system containing the HSV-1 KOS genome was used to generate recombinant viruses as previously described (51). The gI mutants with a HA epitope inserted at the different indicated locations were generated by replacing the WT gI coding sequence with the HA-insertion sequences, which were generated by insertion mutagenesis based on a eukaryotic plasmid for HSV-1 KOS gI, in the background of wild-type KOS or gBsyn BAC system (an A855V mutation was introduced into the gB gene to generate a syncytial virus). All clones were verified by HindIII digestion, PCR analysis, and DNA sequencing of the gI-coding region. Afterward, the BAC plasmids were purified from *E.Coli* (SW102) and transfected into Vero cells with Lipofectamine 2000. After 3 to 4 days posttransfection, when cytopathic effects appeared, the cells were harvested and used to infect new Vero cells to produce a viral stock.

To construct the gI-deletion mutant of HSV-1 McKrae or F strain, we used CRISPR/CAS9 technology by following the method as described previously (52). Briefly, sgRNAs targeting gI were designed using online CRISPR tool and cloned into the plasmid PX335 (sgRNA/Cas9 expression vector) followed by DNA sequencing. The donor GFP flanked by the homologous arms was generated by overlapping PCR, purified, and then cotransfected into Vero CCL81 cells with extracted HSV-1 genome DNA and two sgRNA plasmids using the lipofectamine R 2000 Reagent (Invitrogen, Carlsbad, CA, United States). The cytopathic effect was monitored daily and the recombinant virus carrying GFPwas harvested after 72 h later. The virus was purified by plaque purification with homogeneity monitored by the plaque sizes and confirmed by DNA sequencing. To generate the gI virus, the GFP was replaced by a donor DNA sequence containing the same upstream and downstream homologous arms from acceptor HSV-1. The recombinant virus was generated via transfection and screened by loss of GFP fluorescence.

### Immunofluorescence assay.

Vero cells were grown on coverslips in six-well plates and infected with HSV-1 at an MOI of 5 when the cell confluence reached 60–70%. At indicated time points postinfection, the cells were fixed with 3.7% paraformaldehyde for 10 min at room temperature, then washed with phosphate-buffered saline (PBS) three times (5 min for each wash), permeabilized with PBS containing 0.2% Triton X-100 and 2% bovine serum albumin (BSA) for 10 min, and then blocked with 2% BSA-PBS (2% bovine serum albumin in PBS) mixed with 20% pig serum for 30 min to inhibit nonspecific antibody binding. The cells were then incubated with the proper primary antibodies for 1 h in a humid chamber and then washed with PBS three times (5 min for each wash). After that, the cells were incubated with the appropriate secondary antibodies, including Alexa Fluor 568-conjugated goat anti-rabbit IgG(H+L) F(ab’)_2_ fragment and Alexa Fluor 488-conjugated goat anti-mouse IgG(H+L) F(ab’)_2_ fragment, for another 1 h and then washed once with PBS. All the primary and secondary antibodies were used at a dilution of 1:1,000. Nuclear DNA was stained with 4’,6-diamidino-2-phenylindole (DAPI) (Molecular Probes) for 5 min. After three rinses, the samples were mounted and examined with a Nikon A1 confocal microscope and processed using ImageJ software.

### Immunoprecipitation.

Vero cells seeded in six-well plates were mock-infected or infected with the indicated viruses at an MOI of 5. At 24 h postinfection, the cells were harvested and lysed in NP-40 lysis buffer (0.5% NP-40, 150 mM NaCl, 50 mM Tris-HCl pH 8.0) containing protease inhibitors cocktail (Sigma, catalog no. P8340) on ice. After centrifugation at 12,000 rpm for 20 min, the supernatants were collected and precleared with protein A/G Sepharose beads (Santa Cruz Biotechnology, catalog no. sc-2003) and then incubated with 5 μL indicated antibodies and 25 μL protein A&G Sepharose beads overnight at 4°C with gentle rotation. The beads were washed five times with the NP-40 lysis buffer, and the proteins bounded to the beads were separated by SDS-PAGE, followed by Western blotting analysis.

### Western blotting.

The protein samples were separated by SDS-PAGE, transferred onto polyvinylidene difluoride (PVDF) membranes, blocked with PBST (PBS with 0.05% Tween 20) containing 5% milk or BSA for 1.5 h, and then probed with indicated primary antibodies at RT for 2 h. The membranes were washed with PBST and incubated with appropriate horseradish peroxidase (HRP)-conjugated secondary antibodies with a dilution ratio of 1:10,000 at RT for 1 h. The membranes were again washed and then developed with the Pierce ECL Western blot substrate (Thermo Fisher, catalog no. 32209).

### Virion packaging assay.

The extracellular virions were purified and measured as previously described (14). Briefly, Vero cells were infected with the indicated viruses at an MOI of 5. At 18 hpi, the medium supernatant was separated, centrifuged at 6,000 rpm 4°C for 30 min to remove debris, and next centrifuged in 30% sucrose cushion at 26,000 rpm for 2 h in a Beckman SW32 rotor at 4°C. The precipitates were resuspended in 2 mL NTE buffer (10 mM Tris·HCl, pH 7.4, 100 mM NaCl, 1 mM EDTA) and centrifuged with 30%, 45%, 65% sucrose density gradient at 30,000 rpm for 2h in a Beckman SW41 rotor at 4°C. The purified pelleted virions were resuspended in sample buffer. Both the virions and the cell lysate samples were resolved in 12% SDS-PAGE gel prior to analysis by Western blotting with antibodies against the specified viral proteins.

### Deglycosylation assay.

The Vero cells grown in six-well plates were infected and mock-infected with indicated viruses at an MOI of 5. At 24 h postinfection, the cells were harvested and lysed in NP-40 lysis buffer (0.5% NP-40, 150 mM NaCl, 50 mM Tris-HCl pH 8.0) containing protease inhibitors cocktail (Sigma, catalog no. P8340) on ice. After clarifyied by centrifugation at 12,000 rpm for 20 min, the supernatants were collected and treated with PNGase F or O-glycosidase (New England Biolabs, catalog no. P0733S and P0704S) according to the manufacturer’s instructions. Briefly, the samples were boiled in 1X glycoprotein denaturation buffer (containing 0.5% SDS, 40 mM DTT) at 100°C for 10 min, and then incubated at 37°C in 1X Glycobuffer 2 reaction buffer with 1% NP-40. Afterward, thermal inactivation at 75°C for 10 min. Samples were denatured in SDS loading buffer and subjected to Western blot analysis.

## References

[1] Hill JM, Wen R, Halford WP. 1998. Pathogenesis and molecular biology of HSV latency and ocular reactivation in the rabbit. Methods Mol Med 10:291–315. doi:10.1385/0-89603-347-3:291. 21374237

[2] Johnston C, Koelle DM, Wald A. 2014. Current status and prospects for development of an HSV vaccine. Vaccine 32:1553–1560. doi:10.1016/j.vaccine.2013.08.066. 24016811 PMC4106293

[3] Leung AKC, Barankin B. 2017. Herpes labialis: an update. Recent Pat Inflamm Allergy Drug Discov 11:107–113. doi:10.2174/1872213X11666171003151717. 28971780

[4] Harris KD. 2019. Herpes simplex virus keratitis. Home Healthc Now 37:281–284. doi:10.1097/NHH.0000000000000791. 31483360

[5] Patoulias D, Gavriiloglou G, Kontotasios K, Tzakri M, Keryttopoulos P, Koutras C. 2017. HSV-1 encephalitis: high index of clinical suspicion, prompt diagnosis, and early therapeutic intervention are the triptych of success-report of two cases and comprehensive review of the literature. Case Rep Med 2017:5320839. doi:10.1155/2017/5320839. 28900443 PMC5576427

[6] James SH, Kimberlin DW. 2015. Neonatal herpes simplex virus infection. Infect Dis Clin North Am 29:391–400. doi:10.1016/j.idc.2015.05.001. 26154662

[7] Harris SA, Harris EA. 2018. Molecular mechanisms for herpes simplex virus type 1 pathogenesis in Alzheimer's disease. Front Aging Neurosci 10:48. doi:10.3389/fnagi.2018.00048. 29559905 PMC5845560

[8] Johnson DC, Huber MT. 2002. Directed egress of animal viruses promotes cell-to-cell spread. J Virol 76:1–8. doi:10.1128/jvi.76.1.1-8.2002. 11739666 PMC135733

[9] Smith G. 2012. Herpesvirus transport to the nervous system and back again. Annu Rev Microbiol 66:153–176. doi:10.1146/annurev-micro-092611-150051. 22726218 PMC3882149

[10] Kramer T, Enquist LW. 2013. Directional spread of alphaherpesviruses in the nervous system. Viruses 5:678–707. doi:10.3390/v5020678. 23435239 PMC3640521

[11] Atanasiu D, Saw WT, Cohen GH, Eisenberg RJ. 2010. Cascade of events governing cell-cell fusion induced by herpes simplex virus glycoproteins gD, gH/gL, and gB. J Virol 84:12292–12299. doi:10.1128/JVI.01700-10. 20861251 PMC2976417

[12] Collins WJ, Johnson DC. 2003. Herpes simplex virus gE/gI expressed in epithelial cells interferes with cell-to-cell spread. J Virol 77:2686–2695. doi:10.1128/jvi.77.4.2686-2695.2003. 12552008 PMC141120

[13] David AT, Baghian A, Foster TP, Chouljenko VN, Kousoulas KG. 2008. The herpes simplex virus type 1 (HSV-1) glycoprotein K(gK) is essential for viral corneal spread and neuroinvasiveness. Curr Eye Res 33:455–467. doi:10.1080/02713680802130362. 18568883

[14] Han J, Chadha P, Starkey JL, Wills JW. 2012. Function of glycoprotein E of herpes simplex virus requires coordinated assembly of three tegument proteins on its cytoplasmic tail. Proc Natl Acad Sci USA 109:19798–19803. doi:10.1073/pnas.1212900109. 23150560 PMC3511771

[15] Johnson DC, Frame MC, Ligas MW, Cross AM, Stow ND. 1988. Herpes simplex virus immunoglobulin G Fc receptor activity depends on a complex of two viral glycoproteins, gE and gI. J Virol 62:1347–1354. doi:10.1128/JVI.62.4.1347-1354.1988. 2831396 PMC253147

[16] Ndjamen B, Farley AH, Lee T, Fraser SE, Bjorkman PJ. 2014. The herpes virus Fc receptor gE-gI mediates antibody bipolar bridging to clear viral antigens from the cell surface. PLoS Pathog 10:e1003961. doi:10.1371/journal.ppat.1003961. 24604090 PMC3946383

[17] Frank I, Friedman HM. 1989. A novel function of the herpes simplex virus type 1 Fc receptor: participation in bipolar bridging of antiviral immunoglobulin G. J Virol 63:4479–4488. doi:10.1128/JVI.63.11.4479-4488.1989. 2552134 PMC251078

[18] Hook LM, Huang J, Jiang M, Hodinka R, Friedman HM. 2008. Blocking antibody access to neutralizing domains on glycoproteins involved in entry as a novel mechanism of immune evasion by herpes simplex virus type 1 glycoproteins C and E. J Virol 82:6935–6941. doi:10.1128/JVI.02599-07. 18480440 PMC2446985

[19] Basu S, Dubin G, Nagashunmugam T, Basu M, Goldstein LT, Wang L, Weeks B, Friedman HM. 1997. Mapping regions of herpes simplex virus type 1 glycoprotein I required for formation of the viral Fc receptor for monomeric IgG. J Immunol 158:209–215. 8977192

[20] Farnsworth A, Goldsmith K, Johnson DC. 2003. Herpes simplex virus glycoproteins gD and gE/gI serve essential but redundant functions during acquisition of the virion envelope in the cytoplasm. J Virol 77:8481–8494. doi:10.1128/jvi.77.15.8481-8494.2003. 12857917 PMC165244

[21] Mijnes JD, Lutters BC, Vlot AC, van Anken E, Horzinek MC, Rottier PJ, de Groot RJ. 1997. Structure-function analysis of the gE-gI complex of feline herpesvirus: mapping of gI domains required for gE-gI interaction, intracellular transport, and cell-to-cell spread. J Virol 71:8397–8404. doi:10.1128/JVI.71.11.8397-8404.1997. 9343196 PMC192302

[22] Polcicova K, Goldsmith K, Rainish BL, Wisner TW, Johnson DC. 2005. The extracellular domain of herpes simplex virus gE is indispensable for efficient cell-to-cell spread: evidence for gE/gI receptors. J Virol 79:11990–12001. doi:10.1128/JVI.79.18.11990-12001.2005. 16140775 PMC1212635

[23] Dingwell KS, Johnson DC. 1998. The herpes simplex virus gE-gI complex facilitates cell-to-cell spread and binds to components of cell junctions. J Virol 72:8933–8942. doi:10.1128/JVI.72.11.8933-8942.1998. 9765438 PMC110310

[24] Wisner T, Brunetti C, Dingwell K, Johnson DC. 2000. the extracellular domain of herpes simplex virus gE is sufficient for accumulation at cell junctions but not for cell-to-cell spread. J Virol 74:2278–2287. doi:10.1128/jvi.74.5.2278-2287.2000. 10666258 PMC111709

[25] Johnson DC, Webb M, Wisner TW, Brunetti C. 2001. Herpes simplex virus gE/gI sorts nascent virions to epithelial cell junctions, promoting virus spread. J Virol 75:821–833. doi:10.1128/JVI.75.2.821-833.2001. 11134295 PMC113978

[26] Watson G, Xu W, Reed A, Babra B, Putman T, Wick E, Wechsler SL, Rohrmann GF, Jin L. 2012. Sequence and comparative analysis of the genome of HSV-1 strain McKrae. Virology 433:528–537. doi:10.1016/j.virol.2012.08.043. 23021301

[27] Zhang W, Gao P, Gui X, Zhou L, Ge X, Guo X, Wills JW, Han J, Yang H. 2020. Induction of rod-shaped structures by herpes simplex virus glycoprotein I. J Virol 94:e00231-20. doi:10.1128/JVI.00231-20. 32581097 PMC7431791

[28] Sullivan V, Smith GL. 1988. The herpes simplex virus type 1 US7 gene product is a 66K glycoprotein and is a target for complement-dependent virus neutralization. J Gen Virol 69:859–867. doi:10.1099/0022-1317-69-4-859. 2833569

[29] Olofsson S, Bolmstedt A. 1998. Use of lectins for characterization of O-linked glycans of herpes simplex virus glycoproteins. Methods Mol Med 9:175–192. doi:10.1385/0-89603-396-1:175. 21374459

[30] Ghosh-Choudhury N, Butcher M, Reid E, Ghosh HP. 1994. Effect of tunicamycin and monensin on the transport to the cell surface and secretion of a viral membrane glycoprotein containing both N- and O-linked sugars. Biochem Cell Biol 72:20–25. doi:10.1139/o94-004. 8068242

[31] Amin I, Vajeeha A, Younas S, Afzal S, Shahid M, Nawaz R, Khan MU, Idrees M. 2019. HSV-1 infection: role of viral proteins and cellular receptors. Crit Rev Eukaryot Gene Expr 29:461–469. doi:10.1615/CritRevEukaryotGeneExpr.2019025561. 32422002

[32] Awasthi S, Friedman HM. 2016. Molecular association of herpes simplex virus type 1 glycoprotein E with membrane protein Us9. Arch Virol 161:3203–3213. doi:10.1007/s00705-016-3028-z. 27568015 PMC5727577

[33] Balan P, Davis-Poynter N, Bell S, Atkinson H, Browne H, Minson T. 1994. An analysis of the in vitro and in vivo phenotypes of mutants of herpes simplex virus type 1 lacking glycoproteins gG, gE, gI or the putative gJ. J Gen Virol 75:1245–1258. doi:10.1099/0022-1317-75-6-1245. 8207391

[34] Chapman TL, You I, Joseph IM, Bjorkman PJ, Morrison SL, Raghavan M. 1999. Characterization of the interaction between the herpes simplex virus type i fc receptor and immunoglobulin G. J Biol Chem 274:6911–6919. doi:10.1074/jbc.274.11.6911. 10066744

[35] Rizvi SM, Raghavan M. 2001. An N-terminal domain of herpes simplex virus type Ig E is capable of forming stable complexes with gI. J Virol 75:11897–11901. doi:10.1128/JVI.75.23.11897-11901.2001. 11689673 PMC114778

[36] Howard PW, Howard TL, Johnson DC. 2013. Herpes simplex virus membrane proteins gE/gI and US9 act cooperatively to promote transport of capsids and glycoproteins from neuron cell bodies into initial axon segments. J Virol 87:403–414. doi:10.1128/JVI.02465-12. 23077321 PMC3536398

[37] Carmichael JC, Wills JW. 2019. Differential requirements for gE, gI, and UL16 among herpes simplex virus 1 syncytial variants suggest unique modes of dysregulating the mechanism of cell-to-cell spread. J Virol 93:e00494-19. doi:10.1128/JVI.00494-19. 31092572 PMC6639296

[38] Meyer F. 2016. Viral interactions with components of the splicing machinery. Prog Mol Biol Transl Sci 142:241–268. doi:10.1016/bs.pmbts.2016.05.008. 27571697

[39] Rutkowski AJ, Erhard F, L'Hernault A, Bonfert T, Schilhabel M, Crump C, Rosenstiel P, Efstathiou S, Zimmer R, Friedel CC, Dölken L. 2015. Widespread disruption of host transcription termination in HSV-1 infection. Nat Commun 6:7126. doi:10.1038/ncomms8126. 25989971 PMC4441252

[40] Sadek J, Read GS. 2016. The splicing history of an mRNA affects its level of translation and sensitivity to cleavage by the virion host shutoff endonuclease during herpes simplex virus infections. J Virol 90:10844–10856. doi:10.1128/JVI.01302-16. 27681125 PMC5110170

[41] Starokadomskiĭ PL. 2007. Protein splicing. Mol Biol (Mosk) 41:314–330. 17514899

[42] Anraku Y, Satow Y. 2009. Reflections on protein splicing: structures, functions and mechanisms. Proc Jpn Acad Ser B Phys Biol Sci 85:409–421. doi:10.2183/pjab.85.409. PMC3621562 19907126

[43] Light S, Elofsson A. 2013. The impact of splicing on protein domain architecture. Curr Opin Struct Biol 23:451–458. doi:10.1016/j.sbi.2013.02.013. 23562110

[44] Romero-Casañas A, Gordo V, Castro J, Ribó M. 2020. Protein splicing: from the foundations to the development of biotechnological applications. Methods Mol Biol 2133:15–29. doi:10.1007/978-1-0716-0434-2_2. 32144661

[45] Dill KA, Shortle D. 1991. Denatured states of proteins. Annu Rev Biochem 60:795–825. doi:10.1146/annurev.bi.60.070191.004051. 1883209

[46] Wang W, Nema S, Teagarden D. 2010. Protein aggregation–pathways and influencing factors. Int J Pharm 390:89–99. doi:10.1016/j.ijpharm.2010.02.025. 20188160

[47] Pack SP, Kang TJ, Yoo YJ. 2013. Protein thermostabilizing factors: high relative occurrence of amino acids, residual properties, and secondary structure type in different residual state. Appl Biochem Biotechnol 171:1212–1226. doi:10.1007/s12010-013-0195-1. 23564432

[48] Dobrov EN, Nikitin NA, Trifonova EA, Parshina EY, Makarov VV, Maksimov GV, Karpova OV, Atabekov JG. 2014. β-structure of the coat protein subunits in spherical particles generated by tobacco mosaic virus thermal denaturation. J Biomol Struct Dyn 32:701–708. doi:10.1080/07391102.2013.788983. 24404770

[49] Griffith IP. 1972. The effect of cross-links on the mobility of proteins in dodecyl sulphate-polyacrylamide gels. Biochem J 126:553–560. doi:10.1042/bj1260553. 5075266 PMC1178411

[50] Wang W, Chen Z, Billiar TR, Stang MT, Gao W. 2013. The carboxyl-terminal amino acids render pro-human LC3B migration similar to lipidated LC3B in SDS-PAGE. PLoS One 8:e74222. doi:10.1371/journal.pone.0074222. 24040206 PMC3769297

[51] Gierasch WW, Zimmerman DL, Ward SL, Vanheyningen TK, Romine JD, Leib DA. 2006. Construction and characterization of bacterial artificial chromosomes containing HSV-1 strains 17 and KOS. J Virol Methods 135:197–206. doi:10.1016/j.jviromet.2006.03.014. 16647145

[52] Ren J, Wang H, Zhou L, Ge X, Guo X, Han J, Yang H. 2020. Glycoproteins C and D of PRV strain HB1201 contribute individually to the escape from Bartha-K61 vaccine-induced immunity. Front Microbiol 11:323. doi:10.3389/fmicb.2020.00323. 32210933 PMC7076175

