# Supplementary material for: Discovery and Characterization of an Aberrant Small Form of Glycoprotein I of Herpes Simplex Virus Type I in Cell Culture
Source: Microbiol Spectr. 2022 Mar 29;10(2):e02659-21. doi: 10.1128/spectrum.02659-21 (PMC9045375; doi:10.1128/spectrum.02659-21)
Supplement: SUPPLEMENTAL FILE 1 — Supplemental material. Download SPECTRUM02659-21_Supp_1_seq9.pdf, PDF file, 1.3 MB [file spectrum02659-21_supp_1_seq9.pdf]

Fig.S1 Recognition of gI-GFP in transfection by ployclonal antibodies to gI

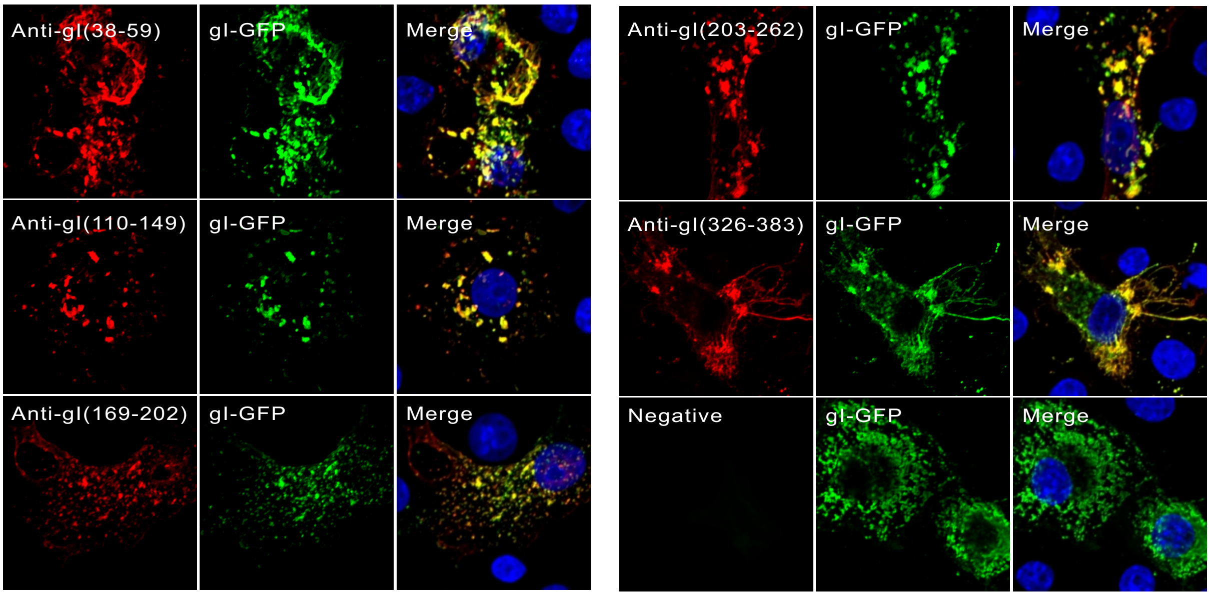

Fig. S1. BHK-21 cells were transfected to express gI-GFP. At 18-24 hours post transfection, the cells were fixed, permeabilized and stained with rabbit peptide antibodies to gI as indicated above or rabbit negative serum. The representative images were captured by a Nikon confocal microscope, and further processed by Image J software.

Fig.S2 Mass spectrometry analysis of the fast-moving species of HSV-1 gI

1 MPCRPLQGLV LVGLWVCATS LVVRGPTVSL VSNSFVDAGA LGPDGVVEED

51 LLILGELRFV GDQVPHTTYY DGVVELWHYP MGHKCPRVVH VVTVTACPRR

101 PAVAFALCRA TDSTHSPAYP TLELNLAQQP LLRVRRATRD YAGVYVLRVW

151 VGDAPNASLF VLGMAIAAEG TLAYNGSAYG SCDPKLLPSS APRLAPASVY

201 QPAPNPASTP STTTSTPSTT IPAPSTTTIPA PQASTTPFPT GDPKPQPPGV

251 NHEPPSNATR ATRDSRYALT VTQIIQIAIP ASIIALVFLG GDPKPQPPGV

301 RRYRRSRRPI YSPQMPTGIS CAVNEAAMAR LGAELKSHPS SCICFIHRCQ

351 RTPMPSLTAI AEESEPAGAA GLPTPPVDPT TPTPTPPLLV

Fig. S2. Vero cells were infected with HSV-1 KOS strain at an MOI of 5. At 24 hpi, the cells were harvested, lysed, and immunoprecipitated with antibody anti-gI (326-383). The imunocomplexes were separated by 12% SDS-PAGE, followed by silver staining. The band corresponding to 23 kDa species was cut off and sent for mass spectrometry analysis. The matched peptides were shown in bold red above.
